# Osteoprotegerin Gene as a Biomarker in the Development of Osteoporosis in Postmenopausal Women

**DOI:** 10.3390/biomedicines11123218

**Published:** 2023-12-04

**Authors:** Filip Przerwa, Izabela Uzar, Anna Bogacz, Katarzyna Kotrych, Tadeusz Sulikowski, Marlena Wolek, Adam Kamiński, Paweł Ziętek, Bogusław Czerny

**Affiliations:** 1 Department of Pharmacology and Pharmacoeconomics, Pomeranian Medical University in Szczecin, 71-230 Szczecin, Poland; fmprzerwa@gmail.com (F.P.); uzari@wp.pl (I.U.); bczerny@wp.pl (B.C.); 2 Department of Personalized Medicine and Cell Therapy, Regional Blood Center, Marcelińska 44, 60-354 Poznan, Poland; 3 Department of General and Dental Radiology, Pomeranian Medical University in Szczecin, al. Powstańców Wielkopolskch 72, 70-111 Szczecin, Poland; kotrych1@gmail.com; 4 General, Mini-Invasive and Gastroenterogical Surgery Clinic, Pomeranian Medical University in Szczecin, 71-252 Szczecin, Poland; tadeusz.sulikowski@pum.edu.pl; 5 Department of Stem Cells and Regenerative Medicine, Institute of Natural Fibres and Medicinal Plants, Kolejowa 2, 62-064 Plewiska, Poland; marlena.wolek@iwnirz.pl; 6 Department of Orthopaedics and Traumatology, Independent Public Clinical Hospital No. 1, Pomeranian Medical University in Szczecin, Unii Lubelskiej 1, 71-252 Szczecin, Poland; emluc@wp.pl; 7 Department of Orthopaedics, Traumatology and Orthopaedic Oncology, Pomeranian Medical University, Unii Lubelskiej 1, 71-252 Szczecin, Poland; pawelziet@gmail.com

**Keywords:** osteoporosis, gene *OPG*, polymorphism, postmenopausal women, osteoprotegerin

## Abstract

Osteoporosis is a multifactorial and polygenic disease caused by an imbalance between osteoclastogenesis and osteoblastogenesis, leading to a decrease in bone mineral density and the occurrence of disorders in the microarchitecture and metabolism of bone tissue. In postmenopausal women, there is a significant decrease in the production of estrogens, which play a key role in maintaining proper bone mineral density. Estrogens have an inhibitory effect on the development and activity of osteoclasts by reducing the synthesis of pro-resorption cytokines and stimulating the expression of osteoprotegerin (OPG). Osteoprotegerin is a cytokine that prevents bone loss by inhibiting the process of osteoclastogenesis, reducing bone resorption. The aim of our study was to determine the influence of the rs3102735 (−163A>G), rs3134070 (−245T>G), rs207361 (−950T>C), rs7844539 (6890A>C), and rs2073618 (1181G>C) polymorphisms of the *OPG* gene on the risk of osteoporosis and osteopenia in postmenopausal Polish women. The study included 802 unrelated women (osteoporosis: *n* = 317, osteopenia: *n* = 110, controls: *n* = 375) at postmenopausal age (54.7 ± 8.6 years). Genetic analysis was performed using real-time PCR. BMD values as well as clinical and bone parameters with the tested polymorphisms were analyzed among the study population. Analysis of the *PPARG* rs1801282 variants did not show any association with the risk of osteoporosis and osteopenia. However, for the *OPG* rs207361 polymorphism, we observed a statistically significant association with the risk of osteoporosis, suggesting that the *OPG* rs207361 variant may be one of the genetic markers associated with the pathogenesis of osteoporosis.

## 1. Introduction

From generation to generation, the average human life expectancy is increasing. This is a result of technological progress, increasing public awareness of health, and, above all, the continuous development of medical science. Increasing life expectancy also brings with it certain difficulties and challenges for modern medicine.

Osteoporosis is a systemic, multifactorial metabolic disease of bone tissue characterized by atrophy of the microarchitecture of bones and a consequent decrease in bone mass. This results in increased fragility and loss of stability and elasticity of the skeleton, leading to fractures, even under low mechanical stress (low-energy injuries). Bone atrophy is caused by a faster breakdown of the bone’s beads and connective tissue relative to bone formation. Osteoporotic fractures significantly reduce the quality of life of patients and are associated with high morbidity, mortality, and economic burden on the health care system [1]. 

Osteoprotegerin (OPG) is a cytokine receptor belonging to the TNF group, but, unlike other TNFs, it lacks a hydrophobic transmembrane domain and is therefore secreted by cells as a soluble protein [2]. Its name comes from its function—to protect bone (*Latin*: “*os*”—bone and “*protegere*”—to protect). OPG is also known as osteoclastogenesis inhibitory factor (OCIF) or TNFRS1 1B, FDRC1, or TR1. OPG’s ligands are RANKL and tumor-necrosis-factor-related apoptosis-activating ligand (TRAIL) [3]. Osteoprotegerin is a key regulator of bone tissue remodeling. It protects bone from excessive resorption by inhibiting the final stages of osteoclastogenesis, suppressing the activation of mature osteoclasts, and further inducing their apoptosis [4]. Both OPG and RANK are receptors that show an affinity for the same ligand, RANKL. OPG is an antagonistic endogenous receptor and, upon binding to RANKL, inhibits osteoclastogenesis, thereby inhibiting the process of bone resorption [5]. This can be confirmed by the fact that transgenic mice in which the *OPG* gene was deleted quickly developed severe osteoporosis. They were observed to have spontaneous fractures due to the excessive formation of the RANKL–RANK complex [6].

The gene encoding *OPG* consists of five exons and is located on the long arm of the eighth chromosome. Osteoprotegerin is synthesized as a propeptide from which a short signal peptide is extracted to form a mature 380 amino acid homodimeric protein that contains seven domains: four cysteine-rich N-terminal domains [1,2,3,7], two apoptosis-related regions [4,8] and a C-terminal heparin-binding region, and a C-terminal heparin-binding domain [5,8].

Due to the role of osteopotegrin in the bone formation process, numerous polymorphisms of the *OPG* gene have become the subject of research regarding their impact on BMD (rs2073618, rs3102735) [9], the risk of osteoporosis (rs3102735, rs2073618) [10], and the response to treatment in postmenopausal osteoporosis (rs3102735, rs2073618) [11]. However, the data are often ambiguous, or even contradictory, and do not include Caucasians. There is a lack of studies focusing on postmenopausal Polish women. The aim of this study was to analyze the selected single-nucleotide polymorphisms (SNPs) of the *OPG* (TNFRSF11B) gene, rs3102735 (−163A>G), rs3134070 (−245T>G), rs207361 (−950T>C), rs7844539 (6890A>C), and rs2073618 (1181G>C), in the development of osteoporosis and osteopenia in postmenopausal women. The polymorphisms selected for investigation are frequently mentioned in the literature as being potentially associated with BMD, osteoporosis, or other bone mineralization disorders.

## 2. Materials and Methods

### 2.1. Study Group 

A total of 802 unrelated postmenopausal Caucasian women in the clinical hospital of the Pomeranian Medical University in Szczecin were enrolled in the study. All patients were informed about the scope of the study before inclusion. The study was approved by the Bioethics Committee of the Pomeranian Medical University in Szczecin (no. KB-0012/100/15). The study was conducted in accordance with the Helsinki Declaration (1975, revised 2000). Written informed consent was obtained from all participants. Patients had their body weight and height measured, which made it possible to calculate body mass index (BMI). The ratios of the subjects’ average bone mineral density to the young adult (YA) average and average bone mineral density to the age-matched average (AM) were also determined.

**Figure 1:**
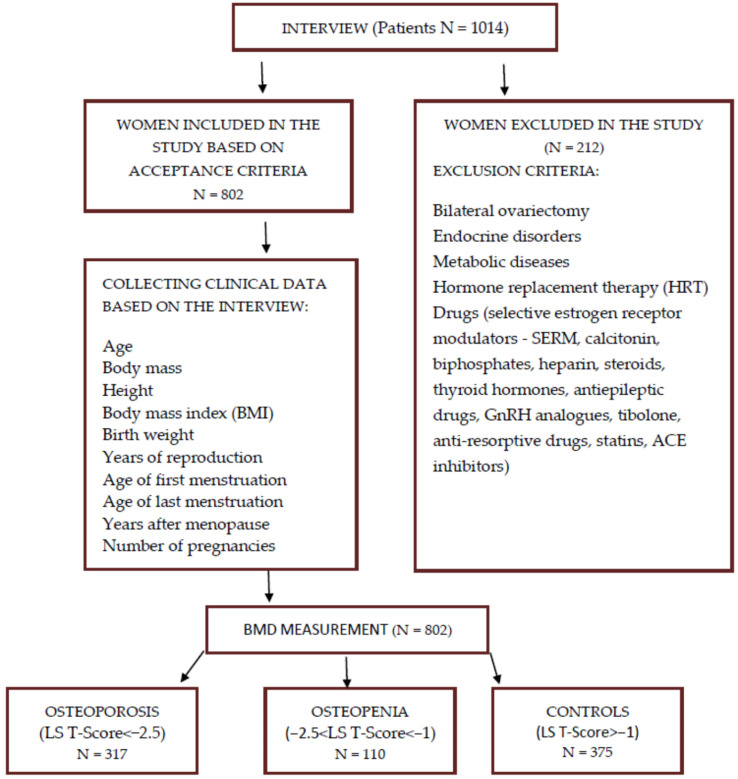
Diagram showing the process of including and excluding participants.

A thorough medical history was taken with the patients qualified to participate in the experiment, including past medical history, birth weight, age of first and last menstruation, and drug treatment used, with particular emphasis on hormonal treatment and preparations from the group of drugs that affect bone mass. Postmenopausal women not taking hormone replacement therapy (HRT) and drugs such as selective estrogen receptor modulators (SERM), calcitonin, biphosphates, heparin, steroids, thyroid hormones, antiepileptic drugs, GnRH analogues, tibolone, anti-resorptive drugs, statins, and ACE inhibitors were eligible for the experiments (Figure 1). Patients with bilateral ovariectomy, endocrine disorders, metabolic diseases, and connective tissue diseases were also excluded from the experiment. Qualified women underwent densitometry using a Lunar DPX100 instrument (Lunar Corp., Madison, WI, USA), with a measurement accuracy of 0.5%. Bone mineral density (BMD) was determined at the lumbar L1–L4 using the dual X-ray beam (DXA) method. The obtained measurements were taken as absolute values (BMD (g/cm^2^)) and related to the mean value in the age group.

The T-score and Z-score values were then calculated, which allowed the assignment of the studied women to different groups. After analyzing the results, measurements that could correspond to degenerative changes in the lumbar spine were excluded from the study. Based on T-score, patients were divided into control group (*n* = 375, T-score > −1.0), women with osteopenia (*n* = 110, T-score between −1.0 and −2), and women with osteoporosis (*n* = 317, T-score < −2.5).

### 2.2. Determination of OPG Polymorphisms

Five *OPG* polymorphisms (rs3102735, rs3134070, rs207361, rs7844539, rs2073618) were analyzed in the development of osteoporosis and osteopenia in postmenopausal women. Real-time polymerase chain reaction (rt-PCR) was used for the analysis. This method uses hybridization probes labeled with a fluorescent dye that binds to DNA at a specific site. With this method, the amount of product in its exponential growth phase can be determined from successive cycle measurements so the DNA amplification process can be monitored in real-time. The LightCycler^®^96 instrument and LightCycler^®^96 Basic Software version 1.2 were used for molecular studies to analyze the results obtained. Fluorescence measured during melting curve analysis was used for genotyping. For polymorphisms, primers and probes specific for the amplified fragments were included—LightSNiP rs3102735, LightSNiP rs3134069, LightSNiP rs2073617, LightSNiP rs7844639, and LightSNiP rs2073618 (TIBMolbiol, Berlin, Germany) reagents were used. Real-time PCR reactions were performed for 45 cycles for each polymorphism tested.

All methods were carried out in accordance with relevant guidelines and regulations.

### 2.3. Statistical Analysis

Statistical analysis of the obtained results was carried out using the R program—version 4.1.2 (R Foundation for Statistical Computing, Vienna, Austria)—and the SNPassoc package. For quantitative variables, an analysis of conformity to the Gaussian distribution was carried out using the Shapiro–Wilk normality test and presented as mean ± standard deviation (SD). In the case of conformity of the trait distribution to the normal distribution, a one-way analysis of variance for unrelated variables (ANOVA) was used to assess the relationship between the means in the study groups, followed by the Tukey HSD post hoc test. Categorical variables were presented as numbers (percentages) and were compared according to the abundance of expected values using Pearson’s χ^2^ test or Fisher’s exact test. All statistical tests performed were two sided. Values of two-sided *p* < 0.05 were considered statistically significant. When the two analyzed characteristics were quantitative, the Pearson correlation with Holm’s correction for multiple testing was used to look for relationships between them.

Pearson’s χ^2^ test was used to check whether each of the studied genetic variants met the assumptions of the Hardy–Weinberg equilibrium (HWE). The associations of the studied SNPs with the risk of osteopenia and osteoporosis were assessed in five genetic models (codominant, dominant, recessive, super-dominant, and log additive) using unconditional logistic regression analysis and presented as odds ratios (OR) and associated 95% confidence intervals (95% CI). Inheritance models were selected using the Akaike information criterion (AIC). 

The linkage disequilibrium (LD) parameters between the studied SNPs and haplotype frequencies were calculated using Haploview version 4.2 software. The linkage disequilibrium analysis was performed for the entire study group. The distance between the analyzed SNPs of the TNFRSF11B gene was calculated. The obtained statistics, Lewontin’s D′, logarithm of the odds (LOD), and correlation coefficients r2, between polymorphic variants are summarized in the Section 3. Associations of TNFRSF11B gene haplotypes with osteopenia and osteoporosis were performed for four polymorphic variants, excluding the rs7844539 variant due to weak coupling with the remaining polymorphisms. Statistically significant *p*-values were corrected using a 10,000-fold permutation test.

## 3. Results

### 3.1. Evaluation of Clinical Data

Table 1 shows the mean densitometric results of female patients in the groups listed in the study. The mean bone mineral density of the lumbar spine (L2–L4), in g/cm^2^, was 1.20 ± 0.10 in the control group, 0.98 ± 0.05 in women with osteopenia, and 0.82 ± 0.07 in patients with osteoporosis (< 0.001). The ratios of mean BMD to the mean for young adult women (YA) and mean BMD to the mean for age (AM) were, respectively, in percentages: 100.45 ± 8.03 and 108.52 ± 10.23 in the control group, 81.71 ± 4.43 and 89.24 ± 6.62 in osteopenia, and 68.26 ± 5.34 and 78.10 ± 7.15 in osteoporosis (*p* < 0.001). The mean values of T-score and Z-score parameters in the control group were 0.05 ± 0.90 and 0.64 ± 1.11, in osteopenia −1.80 ± 0.43 and −0.84 ± 0.66, and −3.16 ± 0.54 and −1.62 ± 0.72 in osteoporosis (*p* < 0.001).

### 3.2. Figures, Tables and Schemes

Analyzing the clinical data collected from medical records and patient interviews, it was observed that the average age of women with osteoporosis was statistically significantly higher than the averages in the other groups. Statistically significant differences were also observed for height, weight, and BMI. The lowest mean height was observed in the group with osteoporosis. Patients with osteoporosis also had the lowest mean body weight, and the difference was statistically significant compared to the mean of women with osteopenia (*p* = 0.011) and the control group (*p* < 0.001). The mean body mass index in the group of women with osteoporosis was significantly lower than the mean in the control group. No differences were observed between the mean years of onset of first and last menstruation and the reproductive period calculated from them. On average, the group with osteoporosis had more years since menopause than the other groups. The number of pregnancies was very similar in all groups. In contrast, the average birth weight of their children was statistically significantly highest for the controls. These results are shown in Table 2.

We also analyzed the association of the patients’ clinical and densitometric data for the different genotypes of the *OPG* gene variants studied in the groups of women separated by T-score. Statistically significant differences were found between the mean neonatal birth weight and genotypes of the rs3134069 in the group of women with osteoporosis and the rs2073618 genotypes in the control group. The mean T-score in patients with osteoporosis differed between genotypes of the rs2073618 polymorphism and averaged 1.21 ± 0.55 for the GG genotype and 1.83 ± 0.61 for GC heterozygotes and 1.61 ± 0.92 for the CC genotype. However, the most interesting results were obtained for rs3102735. For the entire study population of women, the average birth weight of newborns of mothers with the CC genotype was 4056.47 g, which was significantly higher than the average of 3238.33 g of women with the heterozygous TC genotype (*p* < 0.001) and 3177.69 g with the TT genotype. The difference between the averages for the TC and TT genotypes was not statistically significant. After dividing the groups studied in the study, differences were observed for women with osteoporosis and the control group. For patients with osteopenia, the difference was not statistically significant, but the average weight of newborns of mothers with the CC genotype was also the highest and for those with the TT genotype the lowest. Other parameters such as body mass, BMI, and BMD did not show any statistical relationship with the tested polymorphisms.

### 3.3. Evaluation of the Prevalence of the Polymorphisms Studied

After laboratory analysis by real-time PCR, results were obtained for 375 controls, 110 women with osteopenia, and 317 with osteoporosis. The frequencies of the polymorphic variants analyzed in the study followed the Hardy–Weinberg equilibrium (HWE) law for all groups. The Minor Allele Frequency (MAF) for the groups and the percentage of missing genotype designations are shown in Table 3.

### 3.4. Effect of the Prevalence of the Polymorphisms Studied on Osteopenia

Table 4 compares the allele frequencies of the polymorphic variants discussed in the paper in patients with osteopenia and the control group. The frequencies of the rarer alleles of these SNPs were comparable in both groups. Wild-type alleles of variants rs3102735, rs3134069, rs2073617, and rs2073618 were observed more frequently in women with osteopenia compared to the control group. Only for the rs7844539 polymorphism was the rarer C allele present, in 15% of patients with osteopenia and 13% of the control group (OR = 1.23; 95% PU: 0.65–2.34; *p* = 0.526).

In the next stage of the study, we examined the associations of the studied SNPs with the risk of osteopenia in five genetic models (codominant, dominant, recessive, super-dominant, and logarithmic) using unconditional logistic regression analysis. No statistically significant results were obtained for any of the models (Table 5).

### 3.5. Effect of the Prevalence of the Polymorphisms Studied on Osteoporosis

Comparing the allele frequency of the *TNFRSF11B* gene variants studied in the control and osteoporosis groups, a statistically significant difference was observed for rs2073617. The C allele was more common in patients with osteoporosis compared to the control group (51% vs. 46%, *p* = 0.041; OR = 1.249; 95% PU: 1.008–1.548). For the other polymorphic variants, there were no statistically significant differences in allele frequency between the control and osteoporosis groups (Table 6).

Also, in the association analysis of polymorphic variants of the *TNFRSF11B* gene with osteoporosis risk, the most interesting results were obtained for rs2073617. A predominance in the dominant model (TT vs. TC+CC) of genotypes containing the mutant C allele in the group with osteoporosis of 27.3% vs. 22.1% in controls was observed (OR = 1.35; 95% PU: 0.96–1.90; *p* = 0.083; AIC = 930.4). The best model for this variant was the log-additive model (OR = 1.24; 95% PU: 1.00–1.53; *p* = 0.046; AIC = 929.4). For the remaining SNPs, single-locus analysis showed no statistically significant association with osteoporosis (Table 7).

### 3.6. Linkage Disequilibrium Analysis and Haplotype Frequency Analysis of the Analyzed Polymorphisms

**Figure 2:**
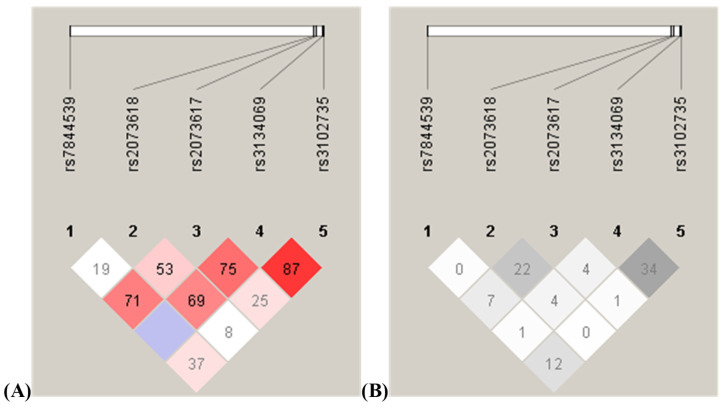
Analysis of coupling disequilibrium between the five SNPs of the *TNFRSF11B* gene analyzed in the study in the entire group of 802 patients. The coupling between SNPs was assessed by D′ and r2 values.(**A**) D′ values are on the left. D′ = 1 indicates full coupling (linkage disequilibrium); D′ = 0 indicates no coupling or coupling balance (linkage equilibrium); (**B**) r2 is on the right side. r2 = 1 means full coupling, r2 > 0.33 means strong coupling, and r2 = 0 coupling equilibrium.

Coupling disequilibrium analysis was performed for the entire study group of 802 women. The distance between the analyzed SNPs of the *TNFRSF11B* gene was only 82 base pairs of the rs3134069 and rs3102735 variants, while rs7844539 and rs3102735 were separated by more than 26 kb (26,345 base pairs, to be exact). Statistics obtained, Lewontin’s D′, the logarithm of the odds (LOD), and r2 correlation coefficients, between polymorphic variants are summarized in Table 8 and shown in Figure 2.

Associations of *TNFRSF11B* gene haplotypes with osteopenia and osteoporosis were carried out for four polymorphic variants. The rs7844539 variant was excluded from the analysis due to its weak association with the other polymorphisms. The obtained associations are shown in Table 9 and the letters in the haplotypes indicate the first allele of the rs2073618 polymorphism, the second allele of the rs2073617 polymorphism, the third allele of the rs3134069 polymorphism, and the fourth allele of the rs3102735 polymorphism, respectively. In the control group, the CTTA haplotype was the most common. The GCTA haplotype was present in 32.4% of patients in the control group and 40.7% of those with osteoporosis (*p* = 0.0078). In contrast, the GTTA haplotype was less frequent in the group of patients with osteoporosis compared to the control group (7.6% vs. 12.1%, *p* = 0.0171). Statistically significant differences were also observed in the frequency of the CCTA haplotype between the control group and osteopenia (*p* = 0.0083) and osteoporosis (*p* = 0.0022) groups. A 10,000-fold permutation test was also performed, which raised the significance level *p*. The *p*-values obtained by this test are shown in Table 9 in parentheses next to the corresponding statistically significant values. The test confirmed statistically significant associations of CCTA and GCTA haplotypes with osteoporosis (*p* = 0.0132 and *p* = 0.0467, respectively).

## 4. Discussion

Osteoporosis is the most common bone disease characterized by decreased bone density and increased risk of fractures. Many studies have shown that genetics is one of the most important risk factors for this disease [12]. The *OPG* gene is one such gene that has been studied for its potential involvement in the etiology of osteoporosis. *OPG* is a protein that regulates bone resorption by inhibiting the activity of osteoclasts, the cells that break down bone tissue. Polymorphisms in the *OPG* gene can alter the expression or function of the *OPG* protein, which can affect bone remodeling and potentially lead to osteoporosis.

The author’s study showed no effect of the rs3102735 polymorphism on the incidence of osteopenia and osteoporosis. This seems to be in line with the study by Wu et al., whose study group was postmenopausal women, with a special focus on women with osteoporosis [13]. They observed that this polymorphism does not affect bone mass either in postmenopausal women with osteoporosis or in healthy postmenopausal women [13]. Similarly, García-Unzueta et al. found no relationship between the rs3102735 polymorphism and reduced bone mass in women [14]. Brambila-Tapia et al. studied the significance of the rs3102735 polymorphism in Mexican women with rheumatoid arthritis and osteoporosis [15]. They did not observe any statistically significant correlations [15]. Similarly, in a Polish study by Boroń et al., where the effect of different *OPG* gene polymorphisms on T-score was investigated, the rs3102735 polymorphism was not found to affect the results of the women studied [16]. The subject of a Brazilian study was the association of serum RANKL and *OPG* levels with vertebral fractures and bone mineral density in women with systemic lupus erythematosus. The data obtained by the researchers did not show statistically significant relationships [17]. On the other hand, the results of a study by - Blascakova et al. showed significant differences between bone mineral density among subjects with AA and GG genotypes (*p* < 0.05) in the control group and AA and AG genotypes (*p* < 0.01) in the osteoporotic group [18]. However, there was no statistical significance in the genotypes represented between the control and osteoporotic groups [18]. Similarly, Abdi et al. found that the rs3102735 polymorphism is not a potential risk factor for postmenopausal osteoporosis but correlates with the BMD of women already suffering from osteoporosis [19].

There are also several studies indicating that the rs3102735 polymorphism is linked to the incidence of osteoporosis. The first was conducted on a Danish population in 2002 by Langdahl et al., and the researchers suggested that the rs3102735 polymorphism may have an impact on both osteoporosis and fracture incidence as the presence of this polymorphism correlated with bone mass [20]. In another 2004 study of Danish postmenopausal women, Jorgensen et al. noted a significant effect of the rs3102735 polymorphism in the *OPG* promoter region on bone mass and fracture incidence, independent of serum osteoprotegerin levels [21]. In 2006, two groups of researchers from China looked at the problem. Hsu et al. found that the GG genotype of the rs3102735 polymorphism positively correlated with total BMD in a population-based study, but this was a study conducted among men [22]. The first Polish study on this topic was conducted in 2009 by Seremak-Mrozikiewicz et al., and 310 unrelated women were included. The researchers concluded that the variant may have a role in the development of osteoporosis in a group of osteoporotic women, but the researchers themselves acknowledged that further research in this direction is needed, with particular attention to the potential influence of other factors [23]. In contrast, a study performed on a group of 200 women in Egypt by Hussien et al. indicated that the homozygous GG genotype occurring with the rs3102735 polymorphism significantly increased the risk of osteoporosis [24]. In 2016, a Croatian study found that postmenopausal women with osteoporosis were more likely to have the AG genotype than women without osteoporosis, which may suggest that the rs3102735 polymorphism influences greater bone mass loss in postmenopausal women. However, as the authors themselves note, the number of subjects they studied was too small to draw firm conclusions [25].

A comprehensive meta-analysis performed in 2022 by Han et al., where they analyzed the results of most of the above studies as well as previously performed meta-analyses, showed that all significant associations between *OPG* rs3102735 polymorphisms and increased osteoporosis risk tended to be false positives, which is in line with the results obtained in this paper [10].

The authors’ study also showed no effect of the rs3134070 gene polymorphism on the incidence of osteoporosis and osteopenia. Similar conclusions were drawn by Wu et al., who examined the relationship between the amount of body fat, the rs3134070 polymorphism, and the incidence of osteoporosis. They noted that the bone mass of postmenopausal women with osteoporosis was significantly lower than that of healthy postmenopausal women. In contrast, the percentage of body fat mass of women with osteoporosis was also significantly lower than that of healthy women. However, the rs3134070 polymorphism alone does not affect BMD in either osteoporotic postmenopausal women or healthy women [26]. Similarly, a study conducted by Kim et al. on a Korean female population showed no effect of this polymorphism on either bone mass or blood osteoprotegerin levels alone [27]. Also, a 2011 study by Mencej-Bedrac et al. showed no effect of rs3134070 on serum OPG levels. This study also showed that this polymorphism cannot be considered a genetic susceptibility factor for postmenopausal osteoporosis and has no effect on bone mineral density [28]. In 2015, Zavala-Cerna et al. studied the osteoprotegerin rs3134070 polymorphism in a Mexican population with rheumatoid arthritis and osteoporosis, finding no statistically significant correlation [29]. Cvijetic et al. also found no effect of this polymorphism on either bone mass or the incidence of osteoporosis [25]. There are also several publications indicating an association of this alteration with osteoporosis and associated fractures. In the case of the rs3134070 polymorphism, the first researchers looking for a correlation between reduced bone mass and osteoporosis were Langdahl et al., who showed that this polymorphism was associated with the incidence of osteoporotic fractures [20]. Other studies showing the effect of this polymorphism on bone mineral density were those by Arko et al. [4] and Zajickova et al. [30]. Of note is the higher prevalence of the rs3134070 polymorphism in systemic lupus erythematosus patients with low BMD compared to patients with normal BMD. Bonfa et al. suggest that a possible explanation is that the G allele may induce *OPG* dysfunction, with adverse effects on BMD, despite normal serum OPG concentrations, or that this polymorphism may have yet another undiscovered effect on bone structure and maintenance of bone health [17].

A meta-analysis conducted in 2022 by Han et al., taking into account all studies conducted to date, also found no effect of the osteoprotegerin rs3134070 polymorphism on the incidence of osteoporosis, which is in line with the conclusions drawn from this study [10].

The study found a statistically significant difference between the incidence of the rs207361 polymorphism in the control group and the osteoporosis group. The C allele was more frequent in patients with osteoporosis compared to the control group. Moreover, in the correlation analysis of polymorphic variants, a predominance of genotypes containing the mutant C allele in the group with osteoporosis of 27.3% vs. 22.1% in controls was observed in the dominant model (TT vs. TC+CC). Similar results were obtained by Wu et al., who found that, in postmenopausal women with osteoporosis, femoral neck BMD was significantly lower in those with the CC genotype compared to those with the TT and TC genotypes. In a group of healthy postmenopausal women, there were no significant differences in BMD between the three genotypes, but the polymorphism itself did not affect the incidence of osteoporosis [31]. In contrast, Boroń et al. showed that the CC genotype may be a factor for the increased risk of faster bone mass loss and osteoporosis in Polish postmenopausal women. The authors suggest that this polymorphism may be a potential genetic marker responsible for the development of osteoporosis [16].

There are also several studies suggesting no association of this polymorphism with osteoporosis. The first report suggesting an effect of this alteration on bones was a Swedish study by Brandstrom et al., where it was suggested to have an effect on reduced bone mass among men [32]. Two years later, Langdahl et al. demonstrated a lack of effect of this polymorphism on both the incidence of osteoporosis and associated fractures. Interestingly, the same study showed that the CC genotype was associated with increased lumbar spine bone mass in osteoporosis patients [20]. However, another study conducted the same year by Wynne et al. found no effect of this polymorphism on bone mass among a population of Irish women [33]. A study by Vidal et al. found no statistically significant association between rs207361 and bone mineral density in postmenopausal women [34]. However, similar to the study by Langdahl et al. [20], CC homozygotes had higher BMD at all anatomical locations, while TT homozygotes had relatively lower BMD [34]. Interestingly, in a study by Zavala-Cerna et al., where the correlation of osteoprotegerin polymorphisms in a Mexican population with rheumatoid arthritis (RA) and osteoporosis was checked, the C allele of the rs207361 polymorphism was shown to be associated with RA, but the main premise of this study was not proven [29]. The lack of an association between rs207361 or its serum concentration and the incidence of osteoporosis in older Australian women was also demonstrated by Ueland et al. [35].

Lee et al. conducted a meta-analysis that included eight studies, and the authors noted no association between rs207361 polymorphisms and bone mineral density [36]. In contrast, the results of a meta-analysis by Guo et al. indicated that rs207361 was associated with the risk of developing osteoporosis [37]. Another meta-analysis on the association of the rs207361 gene polymorphism with osteoporosis risk was conducted by Li et al. and considered the Chinese population. Based on seven studies, which included a total of 1,850 osteoporosis cases and 3074 controls, it showed that the polymorphism was significantly associated with the risk of developing postmenopausal osteoporosis. However, the authors themselves acknowledge that, due to the limitations of this study, the results and conclusions presented should be interpreted with caution [38]. Also, a meta-analysis by Han et al. considering 12 studies, including 1610 cases and 1234 controls, and previous meta-analyses found no statistically significant correlation between the rs207361 polymorphism and osteoporosis [10].

The results of the present study also showed no effect of the rs2073618 polymorphism on the development of osteoporosis. Similarly, Wynne et al. found no significant correlation between lumbar spine BMD and the presence of C alleles [33]. A study by Vidal et al. on the effect of this polymorphism on bone mineral density in postmenopausal women in Malta also did not yield any statistically significant results [34]. Bonfa et al., investigating the effect of rs2073618 on vertebral fractures and bone mineral density in premenopausal women with systemic lupus erythematosus, found no statistically significant relationships [17]. A Mexican study by Nava-Valdivia et al. found no association between rs2073618 and bone mineral density in women with rheumatoid arthritis [39]. A Mexican study by Gonzalez-Mercado et al. that sought to determine the association of this polymorphism with bone mineral density in postmenopausal women also found no association between one and the other. Interestingly, the results of the study indicated that the GG genotype was significantly associated with lower BMI in women with osteoporosis [40]. Both this work and a large number of studies have noted an association of the C allele with higher bone mineral density. A study by Langdahl et al. found that the CC genotype of the rs2073618 polymorphism was less common in patients with osteoporosis and was associated with changes in bone mass at the lumbar spine. Those with the GG genotype had higher BMD at all locations than those with the GC genotype, but, as the authors themselves report, the difference was not statistically significant. Moreover, the polymorphism was not a significant predictor of osteoporotic fractures. These results did not provide conclusive evidence for the role of the rs2073618 polymorphism in the pathogenesis of osteoporosis [20]. Similarly, Choi et al. showed that rs2073618 was associated with the BMD of the radius bone and heel bone in postmenopausal Korean women. The mineral density of these bones in women with the CC genotype was higher than in those with the GG genotype [41]. Interestingly, Moffett et al. also found an association of GG genotype rs2073618 with lower BMD, but, at the same time, there was a lower risk of femoral neck and hip fracture in these individuals [42]. Garcia-Uzueta et al. indicated that the C allele of the rs2073618 polymorphism is associated with higher BMD at the lumbar spine in women. The researchers suggest that it affects the trabecular bone more significantly than the coccyx, as there was no significant association with hip BMD. Moreover, since the relationship is stronger in premenopausal women, it may be more relevant to peak bone mass than to hip BMD [14]. On the other hand, Kim et al., investigating the association between osteoprotegerin, RANK, and RANKL gene polymorphisms and circulating OPG, soluble RANKL, and bone mineral density in Korean postmenopausal women, noted that rs2073618, in combination with the RANKL rs2277438 polymorphism, appeared to increase lumbar spine BMD, suggesting a significant gene–gene interaction between *OPG* and *RANKL* polymorphisms [27].

A study by Arko et al. looked at eight different *OPG* polymorphisms [4]. According to their analysis, the rs2073618 polymorphism is associated with BMD and can therefore be considered a component of genetic susceptibility to osteoporosis [4]. In contrast, the conclusion of a study by Mencej-Bedrac et al. is that this polymorphism can be considered a risk factor for genetic susceptibility to postmenopausal osteoporosis while not affecting serum OPG levels [28]. The results of Boron et al. indicated that this polymorphism is associated with reduced bone density and increased risk of postmenopausal osteoporosis, but also with women’s body weight and birth weight [16]. A study conducted on a population of Arab postmenopausal women by Abdi et al. found a significant difference in the allelic and genotypic distribution of rs2073618 among participants with and without osteoporosis. According to the researchers, the CG heterozygote was associated with a reduced risk of postmenopausal osteoporosis. The G allele had a protective effect against the disease, which is in contrast to the aforementioned study, where it was found that women with osteoporosis were less likely to have the C allele. In addition, in the same study by Abdi et al., the polymorphism analysis showed no effect on either serum OPG, RANKL, or BMD levels [19].

A meta-analysis by Han et al. in 2022, which analyzed most of the above studies, yielding a total of 3472 cases and 3035 controls, showed that the significant results between the rs2073618 gene polymorphism and osteoporosis risk were most likely false positives, so this variation was shown to not affect the incidence of osteoporosis, which is consistent with the results of the present study [10].

The results of this study also indicate a statistically significant association between CCTA and GCTA haplotypes and osteoporosis (*p* = 0.0132 and *p* = 0.0467, respectively). Furthermore, statistically significant differences in the frequency of the CCTA haplotype were observed between the control group and individuals with osteopenia (*p* = 0.0083) and osteoporosis (*p* = 0.0022). A 10,000-fold permutation test confirmed the statistically significant associations of CCTA and GCTA haplotypes with osteoporosis (*p* = 0.0132 and *p* = 0.0467, respectively).

Moreover, it was demonstrated that, in the control group, the most prevalent haplotype was CTTA. The GCTA haplotype occurred in 32.4% of patients in the control group and 40.7% of those with osteoporosis (*p* = 0.0078). Conversely, the GTTA haplotype was less frequent in the osteoporosis group compared to the control group (7.6% vs. 12.1%, *p* = 0.0171). In the literature, results regarding the association of haplotypes with osteoporosis are inconclusive, and various polymorphisms and haplotypes are investigated, and comparing them is difficult.

No association was found of haplotype TNFRSF11B with bone mineral density (BMD) in the femoral neck among the postmenopausal Chinese women examined [43].

The study by Canto-Cetina et al. also did not demonstrate an association of the haplotype TNFRSF11B with bone mineral density (BMD) measured at three skeletal locations [44]. However, Vidal et al., in their study, demonstrated that, in the examined population of Caucasian women, the T-G-T haplotype may increase the risk of osteoporosis due to lower expression of the OPG transcript, leading to increased bone resorption [34].

On the other hand, Rojano-Mejia et al. demonstrated that, in the studied population of Mexican women, the analysis of TNFRSF11B haplotypes serves as a reliable genetic marker for changes in bone mineral density (BMD), with the A-G-T haplotype being associated with alterations in BMD in the femoral neck [45]. The suggestion is made to continue research in other populations due to ethnic diversity [45].

## 5. Conclusions

In conclusion, the study of *OPG* gene polymorphisms (rs3102735, rs3134070, rs207361, rs7844539, and rs2073618) indicated that the rs2073617 polymorphism has a statistically significant effect on the incidence of postmenopausal osteoporosis. The results indicate that there is no relationship between the rs3102735, rs3134069, rs7844639, and rs2073618 polymorphisms and the incidence of postmenopausal osteoporosis. The presence of the C allele of the rs2073618 polymorphism was also found to increase bone mineral density in postmenopausal women. In contrast, the rs2073617 polymorphism may have potential diagnostic significance in monitoring the development of postmenopausal osteoporosis.

A limitation of this study is the small number of patients needed to further validate the results. The strengths of the study concern the detailed analysis of clinical data (e.g., BMI, BMD, birth weight, reproductive years, first and last menstrual period) with the tested polymorphisms and their impact on the risk of osteoporosis. Secondly, the study examined five OPG gene polymorphisms simultaneously, which is rarely seen in other clinical studies.

## Figures and Tables

**Table 1:** Comparison of densitometric findings for female patients.

Parameter	A Control Group (*n* = 375)	B Osteopenia (*n* = 110)	C Osteoporosis (*n* = 317)	*p* (ANOVA)	*p* (Tukey HSD)
BMD L2–L4 (g/cm^2^)				<0.001	C-A–<0.001
*mean ± SD*	1.20 ± 0.10	0.98 ± 0.05	0.82 ± 0.07		B-A–<0.001
*median (min–max)*	1.19 (1.08–1.47)	0.97 (0.90–1.07)	0.83 (0.63–0.90)		B-C–<0.001
BMD L2–L4 YA *(%)*				<0.001	C-A–<0.001
*mean ± SD*	100.45 ± 8.03	81.71 ± 4.43	68.26 ± 5.34		B-A–<0.001
*median (min–max)*	99.00 (90.00–123.00)	81.00 (75.00–89.00)	69.00 (53.00–75.00)		B-C–<0.001
BMD L2–L4 AM *(%)*				<0.001	C-A–<0.001
*mean ± SD*	108.52 ± 10.23	89.24 ± 6.62	78.10 ± 7.15		B-A–<0.001
*median (min–max)*	107.00 (91.00–133.00)	89.00 (74.00–108.00)	78.00 (60.00–92.00)		B-C–<0.001
T-score				<0.001	C-A–<0.001
*mean ± SD*	0.05 ± 0.90	−1.80 ± 0.43	−3.16 ± 0.54		B-A–<0.001
*median (min–max)*	−0.17 (−0.97–3.13)	−1.89 (−2.49)–(−1.05)	−3.05 (-4.73)–(−2.50)		B-C–<0.001
Z-score				<0.001	C-A–<0.001
*mean ± SD*	0.64 ± 1.11	−0.84 ± 0.66	−1.62 ± 0.72		B-A–<0.001
*median (min–max)*	0.56 (−1.85–2.65)	−0.88 (−2.36–0.77)	−1.62 (−3.13–0.98)		B-C–<0.001

**Table 2:** Comparison of clinical data of female patients.

Parameter	A Control Group (*n* = 375)	B Osteopenia (*n* = 110)	C Osteoporosis (*n* = 317)	*p* (ANOVA)	*p* (Tukey HSD)
Patient age (years)				0.007	C-A–0.058
*mean ± SD*	53.49 ± 8.23	53.19 ± 8.20	56.45 ± 8.83		B-A–0.972
*median (min–max)*	55.00 (51.00–71.00)	53.00 (52.00–77.00)	57.00 (51.00–78.00)		B-C–0.010
Height (cm)				<0.001	C-A–0.002
*mean ± SD*	163.17 ± 5.98	162.81 ± 5.02	160.18 ± 5.12		B-A–0.905
*median (min–max)*	164.00 (152.00–180.00)	163.00 (150.00–175.00)	160.00 (150.00–175.00)		B-C–0.002
Body mass (kg)				<0.001	C-A–<0.001
*mean ± SD*	68.71 ± 12.22	65.51 ± 11.14	60.93 ± 9.16		B-A–0.148
*median (min–max)*	66.00 (50.00–100.00)	65.00 (41.00–114.00)	61.00 (43.00–85.00)		B-C–0.011
BMI (kg/m^2^)				0.004	C-A–0.003
*mean ± SD*	25.84 ± 4.55	24.72 ± 3.97	23.70 ± 3.10		B-A–0.157
*median (min–max)*	24.76 (18.33–37.18)	24.98 (17.30–43.43)	23.63 (17.10–31.63)		B-C–0.168
First menstruation (years)				0.654	C-A–0.630
*mean ± SD*	13.38 ± 1.88	13.12 ± 2.39	12.94 ± 2.16		B-A–0.854
*median (min–max)*	14.00 (9.00–16.00)	13.00 (9.00–18.00)	13.00 (9.00–18.00)		B-C–0.894
Last menstruation (years)				0.057	C-A–0.060
*mean ± SD*	50.17 ± 4.39	49.34 ± 4.59	48.06 ± 5.08		B-A–0.646
*median (min–max)*	50.00 (41.00–58.00)	50.00 (38.00–60.00)	49.00 (34.00–60.00)		B-C–0.237
Reproductive period *(years)*				0.724	C-A–0.766
*mean ± SD*	36.38 ± 5.35	36.20 ± 4.93	35.62 ± 5.01		B-A–0.986
*median (min–max)*	37.00 (27.00–48.00)	36.50 (23.00–49.00)	36.00 (24.00–48.00)		B-C–0.795
Years since menopause				0.001	C-A–0.013
*mean ± SD*	7.03 ± 5.59	7.18 ± 6.02	10.63 ± 5.75		B-A–0.992
*median (min–max)*	6.50 (1.00–23.00)	6.00 (0.00–25.00)	10.00 (1.00–22.00)		B-C–0.003
Number of pregnancies				0.852	C-A–0.994
*mean ± SD*	1.94 ± 1.22	1.84 ± 1.13	1.92 ± 1.31		B-A–0.867
*median (min–max)*	2.00 (0.00–6.00)	2.00 (0.00–6.00)	2.00 (0.00–7.00)		B-C–0.902
Newborn’s weight (g)				0.005	C-A–0.009
*mean ± SD*	3628.95 ± 480.75	3226.79 ± 411.07	3141.25 ± 536.32		B-A–0.014
*median (min–max)*	3600 (2460–5100)	3200 (2500–4500)	3000 (2470–4500)		B-C–0.828

**Table 3:** SNP analysis for the whole group.

Rsnumber	Control Group *N = 375*			Osteopenia *N = 110*			Osteoporosis *N = 317*		
	MAF	HWE *p*	Missing *(%)*	MAF	HWE *p*	Missing *(%)*	MAF	HWE *p*	Missing (%)
rs3102735	G = 14.2	0.28	1.6	G = 12.6	0.37	1.8	G = 14.9	0.51	0.6
rs3134069	G = 7.3	1.00	3.7	G = 5.3	1.00	4.6	G = 6.6	0.45	15.8
rs2073617	C = 45.9	0.40	2.4	C = 43.2	0.16	5.5	T = 48.5	0.57	2.8
rs2073618	C = 45.8	0.25	1.1	C = 44.4	0.56	1.8	C = 45.4	0.31	0.9
rs7844539	C = 12.9	0.31	1.1	C = 15.4	1.00	1.8	C = 14.0	1.00	2.86

**Table 4:** Allele frequency of *TNFRSF11B* gene polymorphisms in control and osteopenia groups.

SNP	Group	Alleles		Percentage of People Surveyed	Chi^2^	Pearson’s *p*	OR (95% CI)
*163A>G* rs3102735		*A*	*G*	0.98	0.360	0.548	0.87 (0.55–1.37)
	osteopenia	187 (0.87)	27 (0.13)				
	control	633 (0.86)	105 (0.14)				
*245T>G* rs3134069		*T*	*G*	0.96	1.061	0.302	0.70 (0.36–1.38)
	osteopenia	197 (0.95)	11 (0.05)				
	control	669 (0.93)	53 (0.07)				
*950T>C* rs2073617		*T*	*C*	0.97	0.472	0.491	0.90 (0.66–1.22)
	osteopenia	117 (0.57)	89 (0.43)				
	control	396 (0.54)	336 (0.46)				
*1181G>C* rs2073618		*G*	*C*	0.99	0.136	0.711	0.94 (0.70–1.28)
	osteopenia	119 (0.56)	95 (0.44)				
	control	402 (0.54)	340 (0.46)				
*6890A>C* rs7844539		*A*	*C*	0.98	0.401	0.526	1.23 (0.65–2.34)
	osteopenia	181 (0.85)	33 (0.15)				
	control	648 (0.87)	96 (0.13)				

**Table 5:** Association of *TNFRSF11B* gene variants with osteopenia.

SNP	Model	Control	Osteopenia	OR (95% PU)	*p*	AIC
*163A>G* rs3102735	*AA*	274 (74.3)	83 (77.6)	1.00	0.752	512.8
	*AG*	85 (23.0)	21 (19.6)	0.82 (0.48–1.40)		
	*GG*	10 (2.7)	3 (2.8)	0.99 (0.27–3.68)		
	dominant	95 (25.7)	24 (22.4)	0.83 (0.50–1.39)	0.482	510.8
	recessive	359 (97.3)	104 (97.2)	1.04 (0.28–3.83)	0.958	511.3
	overdominant	284 (77.0)	86 (80.4)	0.82 (0.48–1.39)	0.451	510.8
	log additive	369 (77.5)	107 (22.5)	0.88 (0.57–1.36)	0.557	511.0
*245T>G* rs3134069	*TT*	310 (85.9)	93 (89.4)	1.00	0.704	498.6
	*TG*	49 (13.6)	11 (10.6)	0.75 (0.37–1.50)		
	*GG*	2 (0.6)	0 (0.0)	―		
	dominant	51 (14.1)	11 (10.6)	0.72 (0.36–1.44)	0.337	497.4
	recessive	359 (99.4)	104 (100.0)	―	1.000	497.3
	overdominant	312 (86.4)	93 (89.4)	0.75 (0.38–1.51)	0.413	497.6
	log additive	361 (77.6)	104 (22.4)	0.70 (0.36–1.38)	0.704	497.2
*950T>C* rs2073617	*TT*	111 (30.3)	37 (35.9)	1.00	0.504	498.4
	*TC*	174 (47.5)	43 (41.7)	0.74 (0.45–1.22)		
	*CC*	81 (22.1)	23 (22.3)	0.85 (0.47–1.54)		
	dominant	255 (69.7)	66 (64.1)	0.78 (0.49–1.23)	0.284	496.6
	recessive	285 (77.9)	80 (77.7)	1.01 (0.60–1.71)	0.966	497.8
	overdominant	192 (52.5)	60 (58.3)	0.79 (0.51–1.23)	0.297	496.7
	log additive	366 (78.0)	103 (22.0)	0.90 (0.67–1.22)	0.505	497.3
*1181G>C* rs2073618	*GG*	103 (27.8)	31 (29.0)	1.00	0.920	514.2
	*GC*	196 (52.8)	57 (53.3)	0.97 (0.59–1.59)		
	*CC*	72 (19.4)	19 (17.8)	0.88 (0.46–1.67)		
	dominant	268 (72.2)	76 (71.0)	0.94 (0.59–1.52)	0.807	512.3
	recessive	299 (80.6)	88 (82.2)	0.90 (0.51–1.57)	0.700	512.2
	overdominant	175 (47.2)	50 (46.7)	1.02 (0.66–1.57)	0.936	512.3
	log additive	371 (77.6)	107 (22.4)	0.94 (0.68–1.29)	0.702	512.2
*6890A>C* rs7844539	*AA*	282 (75.8)	76 (71.0)	1.00	0.795	227.7
	*AC*	84 (22.6)	29 (27.1)	1.28 (0.61–2.67)		
	*CC*	6 (1.6)	2 (1.9)	1.24 (0.11–14.02)		
	dominant	90 (24.2)	31 (29.0)	1.28 (0.62–2.61)	0.499	225.7
	recessive	366 (98.4)	105 (98.1)	1.16 (0.10–13.08)	0.903	226.1
	overdominant	288 (77.4)	78 (72.9)	1.27 (0.61–2.65)	0.513	225.7
	log additive	372 (36.7)	107 (63.3)	1.24 (0.64–2.38)	0.518	225.7

**Table 6:** Allele frequency of *TNFRSF11B* gene polymorphisms in the control and osteoporosis groups.

SNP	Group	Alleles		Percentage of People with Non-Missing Genotypes	Chi^2^	Pearson’s *p*	OR (95% CI)
*163A>G* rs3102735		*A*	*G*	0.988	0.131	0.717	1.057 (0.782~1.428)
	osteoporosis	536(0.85)	94(0.15)				
	control	633(0.86)	105(0.14)				
*245T>G* rs3134069		*T*	*G*	0.918	0.155	0.693	0.894 (0.514~1.556)
	osteoporosis	508(0.93)	36(0.07)				
	control	669(0.93)	53(0.07)				
*950T>C* rs2073617		*T*	*C*	0.973	4.139	0.041	1.249 (1.008~1.548)
	osteoporosis	299 (0.49)	317 (0.51)				
	control	396 (0.54)	336 (0.46)				
*1181G>C* rs2073618		*G*	*C*	0.989	0.026	0.870	0.982 (0.793~1.216)
	osteoporosis	343(0.55)	285(0.45)				
	control	402(0.54)	340(0.46)				
*6890A>C* rs7844539		*A*	*C*	0.962	0.100	0.750	1.101 (0.606~2.002)
	osteoporosis	505 (0.86)	83 (0.14)				
	control	108 (0.87)	16 (0.13)				

**Table 7:** Associations of *TNFRSF11B* gene variants with osteoporosis.

SNP	Model	Control	Osteoporosis	OR (95% PU)	*p*	AIC
*163A>G* rs3102735	*AA*	274 (74.3)	226 (71.7)	1.00	0.358	947.9
	*AG*	85 (23.0)	84 (26.7)	1.20 (0.84–1.70)		
	*GG*	10 (2.7)	5 (1.6)	0.61 (0.20–1.80)		
	dominant	95 (25.7)	89 (28.3)	1.14 (0.81–1.59)	0.461	947.4
	recessive	359 (97.3)	310 (98.4)	0.58 (0.20–1.71)	0.312	946.9
	overdominant	284 (77.0)	231 (73.3)	1.21 (0.86–1.72)	0.273	946.8
	log additive	369 (53.9)	315 (46.1)	1.06 (0.78–1.43)	0.718	947.8
*245T>G* rs3134069	*TT*	310 (85.9)	119 (87.5)	1.00	0.846	589.0
	*TG*	49 (13.6)	16 (11.8)	0.85 (0.47–1.55)		
	*GG*	2 (0.6)	1 (0.7)	1.30 (0.12–14.50)		
	dominant	51 (14.1)	17 (12.5)	0.87 (0.48–1.56)	0.635	587.1
	recessive	359 (99.4)	135 (99.3)	1.33 (0.12–14.78)	0.820	587.3
	overdominant	312 (86.4)	120 (88.2)	0.85 (0.46–1.55)	0.590	587.0
	log additive	361 (72.6)	136 (27.4)	0.90 (0.52–1.55)	0.693	587.2
*950T>C* rs2073617	*TT*	111 (30.3)	75 (24.4)	1.00	0.135	931.4
	*TC*	174 (47.5)	149 (48.4)	1.27 (0.88–1.83)		
	*CC*	81 (22.1)	84 (27.3)	1.53 (1.01–2.34)		
	dominant	255 (69.7)	233 (75.6)	1.35 (0.96–1.90)	0.083	930.4
	recessive	285 (77.9)	224 (72.7)	1.32 (0.93–1.88)	0.122	931.0
	overdominant	192 (52.5)	159 (51.6)	1.03 (0.76–1.40)	0.829	933.3
	log additive	366 (54.3)	308 (45.7)	1.24 (1.00–1.53)	0.046	929.4
*1181G>C* rs2073618	*GG*	103 (27.8)	89 (28.3)	1.00	0.985	950.8
	*GC*	196 (52.8)	165 (52.5)	0.97 (0.69–1.38)		
	*CC*	72 (19.4)	60 (19.1)	0.96 (0.62–1.50)		
	dominant	268 (72.2)	225 (71.7)	0.97 (0.70–1.36)	0.866	948.8
	recessive	299 (80.6)	254 (80.9)	0.98 (0.67–1.44)	0.921	948.9
	overdominant	175 (47.2)	149 (47.5)	0.99 (0.73–1.34)	0.941	948.9
	log additive	371 (54.2)	314 (45.8)	0.98 (0.79–1.22)	0.866	948.8
*6890A>C* rs7844539	*AA*	282 (75.8)	217 (73,8)	1.00	0.948	290.4
	*AC*	84 (22.6)	71 (24.2)	1.09 (0.55–2.15)		
	*CC*	6 (1.6)	6 (2.0)	1.30 (0.14–11.89)		
	dominant	90 (24.2)	77 (26.0)	1.10 (0.57–2.14)	0.773	288.5
	recessive	366 (98.4)	288 (98.0)	1.27 (0.14–11.59)	0.828	288.5
	overdominant	288 (77.4)	223 (76.0)	1.08 (0.55–2.13)	0.821	288.5
	log additive	372 (54.0)	294 (46.0)	1.10 (0.61–2.00)	0.750	288.4

**Table 8:** Statistics of linkage disequilibrium strength between the analyzed variants of the *TNFRSF11B* gene.

SNP1	SNP2	D′	LOD	r^2^	Distance in bp
rs7844539	rs2073618	0.193	0.52	0.008	25,327
rs7844539	rs2073617	0.712	6.47	0.077	25,558
rs7844539	rs3134069	1.0	1.08	0.01	26,263
rs7844539	rs3102735	0.373	8.5	0.124	26,345
rs2073618	rs2073617	0.539	42.57	0.224	231
rs2073618	rs3134069	0.696	4.89	0.043	936
rs2073618	rs3102735	0.089	0.18	0.001	1018
rs2073617	rs3134069	0.751	9.3	0.048	705
rs2073617	rs3102735	0.254	2.25	0.012	787
rs3134069	rs3102735	0.876	41.12	0.346	82

**Table 9:** Results of association of *TNFRSF11B* gene haplotypes with osteopenia and osteoporosis.

Haplotype *	Group Turnout			Osteopenia vs. Control	Osteoporosis vs. Control
	Control Group	Osteopenia	Osteoporosis	Chi-Squared *p*	Chi-Squared *p*
*CTTA*	0.346	0.380	0.358	0.5531	0.7774
*GCTA*	0.324	0.352	0.407	0.5191	0.0078 (0.0467)
*GTTA*	0.121	0.143	0.076	0.3454	0.0171 (0.0981)
*GTTG*	0.063	0.041	0.062	0.4069	0.8409
*CCTA*	0.055	0.001	0.011	0.0083 (0.0608)	0.0022 (0.0132)
*CCGG*	0.048	0.060	0.064	0.6116	0.3317
*GCTG*	0.014	0.025	0.011	0.8498	0.5419

* rs2073618/rs2073617/rs3134069/rs3102735. In parentheses, the *p*-value after the 10,000-fold permutation test is shown.

## Data Availability

Data are available from the authors upon request.
